# In silico modeling indicates the development of HIV-1 resistance to multiple shRNA gene therapy differs to standard antiretroviral therapy

**DOI:** 10.1186/1742-4690-7-83

**Published:** 2010-10-09

**Authors:** Tanya Lynn Applegate, Donald John Birkett, Glen John Mcintyre, Angel Belisario Jaramillo, Geoff Symonds, John Michael Murray

**Affiliations:** 1 Johnson and Johnson Research Pty Ltd, Level 4 Biomedical Building, 1 Central Avenue, Australian Technology Park, Eveleigh, NSW, 1430, Australia; 2 The National Centre in HIV Epidemiology and Clinical Research, University of New South Wales, Level 9 Lowy Packer Building, 405 Liverpool St, Darlinghurst, NSW, 2010, Australia; 3 9 Raglan St, Mosman, NSW, 2088, Australia; 4 School of Molecular and Microbial Biosciences, School of Biological Sciences, University of Sydney, NSW, 2006, Australia; 5 Cell and Molecular Therapies, Royal Prince Alfred Hospital Missenden Road, Camperdown, NSW, 2050, Australia; 6 Faculty of Medicine, Level 8, Lowy Packer Building, 405 Liverpool St, Darlinghurst, NSW, 2010, Australia; 7 School of Mathematics and Statistics, University of New South Wales, Sydney, NSW, 2052, Australia

## Abstract

**Background:**

Gene therapy has the potential to counter problems that still hamper standard HIV antiretroviral therapy, such as toxicity, patient adherence and the development of resistance. RNA interference can suppress HIV replication as a gene therapeutic via expressed short hairpin RNAs (shRNAs). It is now clear that multiple shRNAs will likely be required to suppress infection and prevent the emergence of resistant virus.

**Results:**

We have developed the first biologically relevant stochastic model in which multiple shRNAs are introduced into CD34+ hematopoietic stem cells. This model has been used to track the production of gene-containing CD4+ T cells, the degree of HIV infection, and the development of HIV resistance in lymphoid tissue for 13 years. In this model, we found that at least four active shRNAs were required to suppress HIV infection/replication effectively and prevent the development of resistance. The inhibition of incoming virus was shown to be critical for effective treatment. The low potential for resistance development that we found is largely due to a pool of replicating wild-type HIV that is maintained in non-gene containing CD4+ T cells. This wild-type HIV effectively out-competes emerging viral strains, maintaining the viral *status quo*.

**Conclusions:**

The presence of a group of cells that lack the gene therapeutic and is available for infection by wild-type virus appears to mitigate the development of resistance observed with systemic antiretroviral therapy.

## Introduction

Human Immunodeficiency Virus type 1 (HIV-1) is a positive strand RNA retrovirus that can cause Acquired Immunodeficiency Syndrome (AIDS) resulting in destruction of the immune system. HIV infection is currently treated with Highly Active Anti-Retroviral Therapy (HAART), a combination treatment of 3 or more drugs that significantly reduces viral replication and disease progression [1]. However, these drugs have side-effects and can lead to low patient adherence resulting in viral breakthrough, one of the greatest challenges of today's treatment regimes. In extreme cases, several rounds of low adherence and viral breakthrough can exhaust all regimens and salvage options, rendering HAART ineffective.

RNA interference (RNAi) is a relatively recently discovered mechanism of gene suppression that has received considerable attention for its potential use in gene therapy strategies for HIV (for Reviews see [2-4]). RNAi can be artificially harnessed to suppress targets of choice by engineering short hairpin RNA (shRNA). Sharing structural similarities to natural microRNA, shRNA consists of a short single stranded RNA transcript that folds into a 'hairpin' configuration by virtue of self-complementary regions separated by a short 'loop' sequence. shRNA-based gene therapy is an attractive alternative to HAART as RNAi is specific, highly potent, and is likely to be free of the side-effects associated with HAART. The potency of individual shRNA against HIV has been extensively demonstrated in tissue culture and there are now several hundred identified shRNA targets and verified activities targeting both HIV and host RNA (e.g. CCR5) to inhibit HIV infection (compiled in [5]). Along with Naito *et. al*. [5] and ter Brake *et. al*. [6], our group has contributed a large proportion of these targets which were specifically designed to be highly conserved amongst known viral variants, and selected for their high suppressive activities [7].

While shRNA is known to be an effective tool to regulate gene expression, the efficacy of single shRNAs in treating HIV infection is limited due to the rapid development of resistance in the target region [8-12]. Many groups, including our own, have studied the feasibility and efficacy of expressing multiple anti-HIV shRNAs to minimize the development of resistance. While it has not yet been demonstrated, the use of multiple shRNAs may also improve anti-viral efficacy by targeting several genes that are critical to distinct stages in the HIV replication cycle. Despite the large replication and error rate, certain viral sequences are faithfully maintained during replication. These highly conserved regions offer excellent targets as they are likely to be critical for viral fitness. Further, the selection of highly conserved sites ensures the therapy matches the maximum number of viral variants. Mathematical analysis of sequence variation in Clade B assessed combinations of highly active and highly conserved shRNA, previously identified in our laboratory [7], that were designed to cover a broad range of HIV target genes (Mcintyre *et. al*. unpublished data). Our analysis indicates that at least 6 highly conserved shRNAs are required to ensure that 100% of Clade B patients will have complete homology to at least 4 of these shRNAs.

Gene therapy is an emerging technology that has demonstrated clinical efficacy and biological effect in treating diseases such as severe combined immune deficiencies (SCID-X1, ADA-SCID) [13,14] and chronic granulomatous disease (CGD) [15], and our own HIV study has demonstrated safety, persistence of gene-containing cells and a biological effect as detailed below [16]. In these cases, the procedure uses a viral vector to deliver a nucleic acid sequence to a HSC target cell that will either restore the activity of impaired gene products or down-regulate a disease causing gene. Autologous CD34+ HSC serve as ideal target cells for gene therapy, as once re-infused, they can differentiate into all hematopoietic lineages, including T cells, granulocytes and macrophages [17]. As they are stem cells, they are capable of providing a continual source of progeny cells containing the therapeutic sequence.

Mathematical modelling of gene therapy has been limited and has mostly considered the average response over time of frequent and predictable events such as CD4+ T cell numbers and HIV viral load [18-20]. Despite providing only a relatively small number of gene-containing cells, our own modelling predicted that HSC gene therapy which prevents HIV entry or integration can have a clinically relevant impact on CD4+ cell counts and viral load [20]. This prediction has been verified by our group in the only randomized, placebo-controlled and double-blinded phase II clinical trial of HIV gene therapy to report its results to date. This trial involved the use of a retroviral vector delivering a *tat/vpr *specific anti-HIV ribozyme (OZ1) in autologous HSC [21]. Over 100 weeks, while the primary viral load endpoint was not significantly different, certain predetermined measures of viral loads (secondary end points) including time-weighted area under the viral load curve were significantly (p < 0.05) different in the OZ1 group compared to placebo: lower log time-weighted area under the viral load curve weeks 40-48 and 40-100; longer time to reach 10, 000 HIV-1 copies/ml; greater number of subjects with plasma viral load of less than 10, 000 copies/ml at weeks 47/48; lower median plasma viral load in the OZ1 subjects who continued to display OZ1 expression beyond week 48. There were also positive trends in viral load at week 48, time to reinitiate HAART, and CD4 and CD8 counts. This study provided the first indication that cell-delivered gene transfer is safe and biologically active in the setting of HIV.

In that phase II study [21], there was modest efficacy with no evidence for the development of viral resistance during the trial period. However, it remains possible that increases in gene therapy efficacy may lead to the development of resistance and reduce durable suppression of viral replication, even with the inclusion of multiple agents. Leonard *et al. *[22] investigated the development of resistance to gene therapy through a stochastic model. Although it provided valuable information about the relationship between multiple RNAi effectors and treatment efficacy, all scenarios assumed that 75 - 100% of CD4+ T cells contained the gene at baseline. (We refer here and throughout this manuscript to such gene-containing cells as transduced or Tx cells). Without prior immune ablation, this is a large and perhaps unobtainable number of gene-containing T cells. As shRNA delivery to HSC would commence with 0% Tx CD4+ T cells, the dynamics of the production of these cells is likely an important factor for the development of resistance during the initial phases of gene therapy. Thus, we developed a stochastic model that specifically addressed the expansion of gene-containing progeny CD4+ T cells from a population of transduced HSC and also included many of the features of the model developed by Leonard *et al. *[22]. It is important to note that unless the patient undergoes hematopoietic ablation, it is to be expected that a sizeable proportion of untransduced (UNTx) CD4+ T cells will always be present regardless of the level of HSC transduction.

The model was developed to determine i) how many shRNAs and ii) their level of inhibition (when delivered to HSC as a gene therapeutic), are required to prevent virological escape. The stochastic model incorporated a 3-dimensional space to represent lymphoid tissue where transmission of HIV is high, and tracked the survival and expansion of individual cells and the evolution of viral sequences in the shRNA targeted region. Using conservative assumptions, we found that combinations of 4 or more shRNAs can stabilize infection at a low level, as long as the shRNAs act prior to integration of pro-viral DNA. Escape mutants did not emerge due to a pool of wild-type (wt) virus replicating in UNTx cells. This wt virus effectively out-competes all emerging mutated strains of reduced fitness. This indicates that gene therapy delivered to HSC can suppress viral load, and can forestall the development of resistance due to a sizeable proportion of cells that do not contain the gene therapeutic. This produces a situation very different to systemic HAART where the drugs are distributed at varying concentrations across all target cells.

## Results

The model was designed to monitor the impact of HSC-delivered gene therapy, in which a combination of non-overlapping shRNAs were expressed, on the development of resistance in a 3-dimensional cube representing lymphoid tissue. The cube contained 70^3 ^(343,000) CD4+ T cells and was followed for 5,000 days, with data collected every 12 hours. Each shRNA was assumed to inhibit both incoming virus prior to integration (Class I) and nascent viral transcripts produced from integrated proviral DNA (Class II); see Methods for a more complete model description. CD34+ HSC were assumed to have been transduced with the gene therapy *ex vivo *and returned to the patient to engraft and to continuously give rise to a supply of gene-containing CD4+ progeny T cells through the thymus [21]. A proportion of all infected cells is long lived to represent latency and maintains a constant source of virus. All non-gene containing progeny CD4+ T cells are referred to as UNTx and gene-containing T cells as Tx cells.

**Figure 1:**
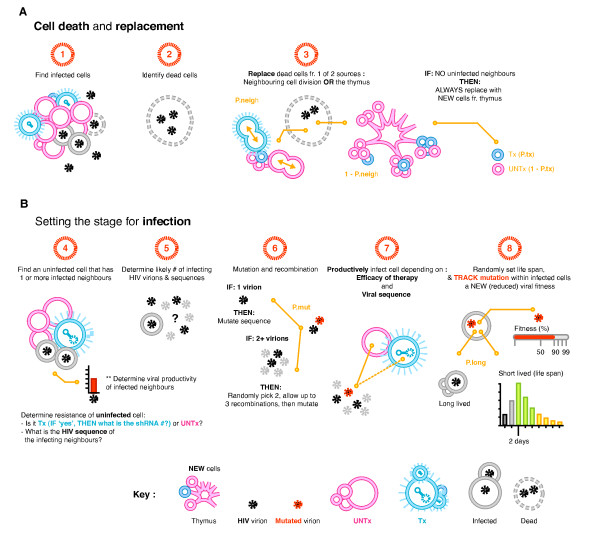
**Key steps, decision points and probabilities of the 3 D stochastic model**. The following parameters were used to determine cell death and replacement, and infection. Cells that do, or do not, contain the integrated gene are referred to as transduced (Tx) or untransduced (UNTx) respectively. Tx or UNTx cells can either be uninfected or infected. (**A**) The replacement of an infected cell is determined by (**1**) finding the infected UNTx or Tx cells, (**2**) identifying the infected dead cells, and (**3**) replacing them with cells divided from uninfected neighbours or newly matured from the thymus (**B**) Infection is established by (**4**) finding an uninfected cell with at least one infected neighbour and determining the protection of the uninfected cell, i.e. is it UNTx or Tx (and with how many shRNAs)? (**5**) The status of the infected neighbour is used to determine the likely number of virions produced and their sequence. (**6**) The virion sequence is mutated and recombined as necessary. (**7**) Cells are infected depending on the infecting viral sequence, any inhibitory shRNA, and chance. (**8**) The life span of the newly infected cell is randomly assigned and the viral fitness is adjusted according to its mutations/recombinations. **Probabilities: P.tx **(set at either 0.2 or 0.01): the percentage of Tx CD34+ hematopoietic stem cells (HSC) resulting in this percentage of cells exported from thymus containing gene product. **P.neigh **(set at 0.99): the replacement by an uninfected neighbour, compared to a cell from the thymus. **P.mut **(set at 3.4 × 10^-5^): the mutation rate per nucleotide. **Viral productivity: **determined by viral fitness, the transduction state of the infected cell (Tx or UNTx) and the number of mutated sequences. **Life span: **Poisson distributed with mean 2 days, measured in 12-hourly intervals. **P.long **(set at 0.0183): probability that an infected cell is long lived.

Each of the scenarios in Table 1 (referred to throughout this manuscript as S1, S2, S3 etc) was initiated with a single wild-type (wt) virus sequence with no mutations in the shRNA target sites, and was pre-run for 100 days to mimic the natural course of infection prior to gene therapy. This enabled HIV to accumulate random mutations and develop into a pool of variant strains to simulate natural HIV diversity. Sequence variation arose randomly with a reverse transcription error rate of 3.4 × 10^-5 ^mutations per HIV RNA nucleotide per round of replication [23]. With this mutation rate and 19 nucleotides for each of the maximum 6 shRNA, 0.39% of infected cells at the start of therapy have a single mutation for the shRNA genes and 0.00074% have double mutations. Hence even in the absence of any selective pressure, all single shRNA mutations (m = 1) and some double mutations (m = 2) will be present before therapy in the simulation of the 343,000 cells. All interactions, described in Figure 1, were governed by chance with an underlying defined probability.

**Table 1:** The scenarios modeled^1^

Scenario (S#)	Class	# shRNA	Efficacy (%)	HSC+ (%)	Fitness (%)
S0	Untreated				
S1	I & II	6	80^n^	20	99
S2	I & II	4	80^n^	20	99
S3	I & II	2	80^n^	20	99
S4	I & II	6	60^n^	20	99
S5	I & II	6	80^n^	1	99
S6	I & II	2	80^n^	20	50
S7	I & II	2	80^n^	1	50
S8	I & II	6	80^n^	20	90
S9	I & II	6	80^n^	20	50
S10	I & II	2	80^n^	20	90
S11	II only	6	80^n^	20	99

^1^Twelve scenarios (S#) varied in the number of shRNAs considered (6, 4 and 2 shRNAs), the efficacy of each shRNA (60 or 80%), the proportion of hematopoietic stem cells transduced with the gene therapeutic (HSC+; 20 or 1%), viral fitness (99, 90, or 50%), and the class of treatment (Class I and II). The untreated control (S0) contained only UNTx cells exposed to HIV.

**Figure 2:**
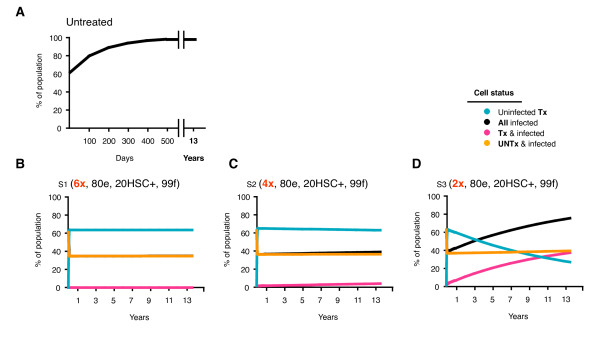
**Increasing the number of shRNAs**. Tx and UNTx, infected and uninfected cells are expressed as a percentage of all cells and monitored over 5000 days. Scenarios include; A) The absence of gene therapy B) 6 shRNAs (S1), C) 4 shRNAs (S2) and D) 2 shRNAs (S3). Assumptions for each scenario include 80^n ^% efficacy (80e), 20% Tx hematopoietic stem cells containing the gene therapeutic (HSC+), 99% fitness (99f) with Class I and II inhibition.

In the absence of gene therapy, the proportion of infected cells increased rapidly and completely saturated the tissue in less than 500 days (Figure 2A). A proportion of these cells harboured new strains, which evolved mutations that would have conferred resistance to 1 (m = 1) or 2 (m = 2) shRNAs in the presence of gene therapy (though no shRNAs were present in this control scenario). The number of cells infected with these mutated strains stabilized at < 1% between 100 and 500 days. These strains thus approximate the diversity within the shRNA target regions expected during the natural course of untreated HIV infection.

### Modeling changes in shRNA number: 6, 4 and 2

**Figure 3:**
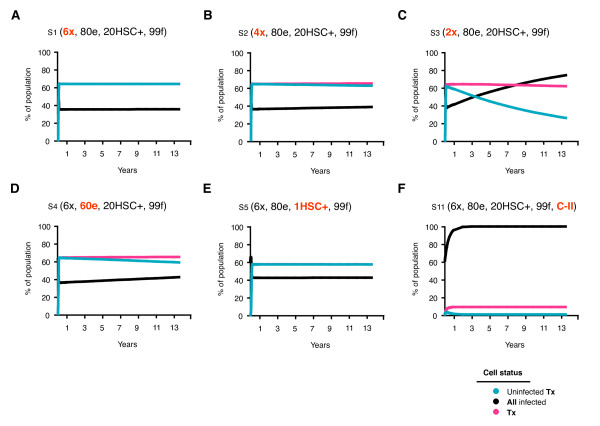
**Effect of the number of shRNA, efficacy, marking and level of inhibition on cellular compartments**. Cells within each compartment are expressed as a percentage of all cells and monitored over 5000 days. Scenarios include: A) 6 shRNAs (S1), B) 4 shRNAs (S2), C) 2 shRNAs (S3), D) 60^n ^% efficacy (S4), E) 1% marking (S5) and F) Class II inhibition only (S11). Assumptions for each scenario are indicated where - x = number of shRNA, - e = efficacy, - HSC+ = hematopoietic stem cells transduced to contain the gene therapeutic and - f = fitness.

The first gene therapy scenarios that we modeled compared the expression of 2 (S3), 4 (S2) and 6 (S1) independent shRNAs (Table 1). These scenarios assumed each shRNA independently inhibited virus by 80%, that 20% of the HSC contained the gene, and mutated virus was 99% fit compared with wt virus. Using these assumptions and those described in the Methods, simulations showed that 2 shRNAs provided inadequate protection (Figure 2D: S3). While uninfected Tx cells accumulated rapidly, this was followed soon after by a steady decline, allowing infected cells to predominate by 2500 days and increase to 74% at 5000 days. In contrast, both 4 and 6 shRNA scenarios allowed uninfected Tx cells to accumulate rapidly and stably constitute > 98% of all uninfected cells (Figure 2B, C: S2 & S1). In these scenarios, a steady state was established quickly with the majority of cells being protected by the shRNAs with essentially no resistant strains emerging (Figure 3: S2 & S1). This protection remained virtually constant through to the end of the simulation at 5000 days and effectively suppressed overall infection to 38% and 35% of all cells respectively (Table 2: S2 & S1).

**Table 2:** Final proportion of each cell population, from comparable scenarios after 5000 days^1^

Scenario	Variable	Uninfected			Infected (percentage of all cells, (SD))				
		UNTx	Tx		UNTx			Tx	
				m = 0	m = 1	m = 2	m = 0	m = 1	m = 2
**shRNA**									

**S1**	**6×**	1.294 (0.024)	63.647 (0.069)	34.608 (0.080)	0.341 (0.028)	0.002 (0.001)	0.102 (0.004)	0.005 (0.001)	0.000 (0.000)
**S2**	**4×**	0.665 (0.017)	61.651 (0.094)	34.901 (0.097)	0.229 (0.023)	0.000 (0.000)	2.474 (0.027)	0.080 (0.010)	0.000 (0.001)
**S3**	**2×**	0.133 (0.021)	25.299 (1.717)	38.049 (0.596)	0.148 (0.026)	0.170 (0.469)	35.210 (0.553)	0.546 (0.085)	0.445 (1.212)
**Efficacy**									
**S1**	**80**	1.294 (0.024)	63.647 (0.069)	34.608 (0.080)	0.341 (0.028)	0.002 (0.001)	0.102 (0.004)	0.005 (0.001)	0.000 (0.000)
**S4**	**60**	0.274 (0.008)	58.296 (0.085)	34.922 (0.085)	0.347 (0.026)	0.002 (0.001)	6.007 (0.044)	0.151 (0.012)	0.002 (0.002)
**HSC+**									
**S1**	**20**	1.294 (0.024)	63.647 (0.069)	34.608 (0.080)	0.341 (0.028)	0.002 (0.001)	0.102 (0.004)	0.005 (0.001)	0.000 (0.000)
**S5**	**1**	0.524 (0.010)	57.043 (0.160)	41.885 (0.170)	0.432 (0.034)	0.003 (0.003)	0.107 (0.004)	0.006 (0.001)	0.000 (0.000)
**S6**	**20**	0.118 (0.004)	26.395 (0.116)	37.835 (0.114)	0.030 (0.005)	0.003 (0.011)	35.501 (0.096)	0.094 (0.015)	0.023 (0.073)
**S7**	**1**	0.216 (0.004)	17.854 (0.160)	48.618 (0.218)	0.045 (0.007)	0.001 (0.004)	33.169 (0.087)	0.093 (0.010)	0.005 (0.016)
**Fitness**									
**S1**	**99**	1.294 (0.024)	63.647 (0.069)	34.608 (0.080)	0.341 (0.028)	0.002 (0.001)	0.102 (0.004)	0.005 (0.001)	0.000 (0.000)
**S8**	**90**	1.296 (0.028)	63.649 (0.092)	34.719 (0.089)	0.231 (0.014)	0.001 (0.001)	0.101 (0.004)	0.003 (0.001)	0.000 (0.000)
**S9**	**50**	1.324 (0.039)	63.689 (0.118)	34.798 (0.138)	0.089 (0.003)	0.000 (0.000)	0.100 (0.005)	0.001 (0.000)	0.000 (0.000)
**S3**	**99**	0.133 (0.021)	25.299 (1.717)	38.049 (0.596)	0.148 (0.026)	0.170 (0.469)	35.210 (0.553)	0.546 (0.085)	0.445 (1.212)
**S10**	**90**	0.124 (0.012)	25.990 (0.439)	37.873 (0.201)	0.098 (0.009)	0.025 (0.057)	35.453 (0.064)	0.360 (0.030)	0.078 (0.184)
**S6**	**50**	0.118 (0.004)	26.395 (0.116)	37.835 (0.114)	0.030 (0.005)	0.003 (0.011)	35.501 (0.096)	0.094 (0.015)	0.023 (0.073)
**Class**									
**S1**	**I & II**	1.294 (0.024)	63.647 (0.069)	34.608 (0.080)	0.341 (0.028)	0.002 (0.001)	0.102 (0.004)	0.005 (0.001)	0.000 (0.000)
**S11**	**II only**	0.001 (0.000)	0.004 (0.001)	90.374 (0.077)	1.059 (0.077)	0.009 (0.008)	8.448 (0.055)	0.105 (0.009)	0.001 (0.001)

^1^Values represent the mean of 10 simulations, expressed as the percentage of all cells (plus standard deviation in brackets). Scenarios (S#) with identical assumptions are grouped under the variable of interest for comparison. For example, the scenarios which have an identical proportion of hematopoietic stem cells transduced with the gene therapeutic (HSC+) are grouped together. The number of shRNAs to which the virus is resistant is denoted as m (for mutations), i.e. m = 0 (wt virus with no mutations in the shRNA target sites), m = 1 (virus with mutations conferring resistance to 1 shRNA), and m = 2 (virus with mutations conferring resistance to 2 shRNAs).

When 2 shRNAs provided inadequate protection, the resistance profile indicated that > 99% of replication was wt and occurred in approximately equal amounts in the UNTx (38%) and Tx (36%) compartments (Table 2: S3). The bulk of viral replication shifted into the UNTx compartment as the number of shRNAs increased, indicating that more than 2 shRNAs were required to provide adequate protection for Tx cells (Figure 2: S3, S2 & S1). Wt virus continued to replicate in the UNTx compartment with an increasing number of shRNAs (38.0 vs. 34.9 vs. 34.6% for 2, 4, and 6 shRNA respectively), though it decreased by more than 2 logs in Tx cells (35.2 vs. 2.5 vs. 0.1% respectively; Table 2: S3, S2 & S1). While the overall number of infected cells decreased with increasing shRNAs, this same selective pressure resulted in a relative increase in resistant virus in the UNTx compartment (e.g. m = 1; 0.148 vs. 0.229 vs. 0.341% for S3, S2 & S1 respectively) and a relative decrease of resistant virus in the Tx compartment (e.g. Table 2: m = 1; 0.546 vs. 0.080 vs. 0.005%, and Figure 3: S3, S2 & S1).

### Modeling changes in shRNA efficacy

shRNA target selection is generally based on i) conservation amongst different viral variants and ii) experimentally determined suppressive activity. We have previously identified suitable anti-HIV shRNAs that are both highly active (> 75% efficacy) and whose target sequence is highly conserved. We used the model to determine if a reduction in shRNA efficacy is likely to affect overall infection or resistance profiles, assuming shRNAs can target both incoming and nascent viral transcripts [24-26]. We simulated a reduction in efficacy of each shRNA from 80% to 60% and kept all other parameters unchanged.

**Figure 4:**
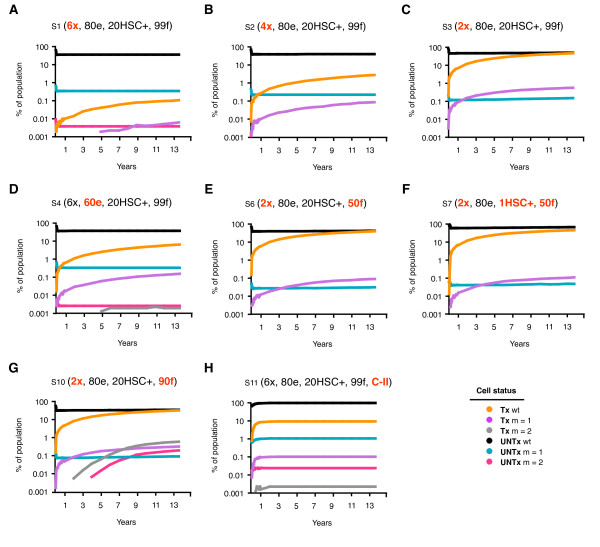
**Effect on the resistance profile in the Tx and UNTx compartments over 5000 days**. The percentage of cells, both Tx and UNTx, infected with virus that is wt, or completely resistant to 1 (m = 1) or 2 (m = 2) shRNA and monitored over 5000 days. Scenarios include A) 6 shRNAs (S1), B) 4 shRNAs (S2) and C) 2 shRNAs (S3), D) 60^n ^% efficacy (S4), E) 50^n ^% fitness (S6), F) 1% marking and 50^n ^% fitness (S7) and G) 90% fitness (S10) and H) Class II inhibition only (S11). Assumptions for each scenario are indicated where - × = number of shRNA, - e = efficacy, - HSC+ = hematopoietic stem cells transduced to contain the gene therapeutic and - f = fitness. Populations that were essentially zero were unable to be plotted on a log scale, and are indicated with an appropriate marker placed at the end of the abscissa.

The reduction in efficacy from 80% (S1) to 60% (S4) led to a slight increase in the number of infected cells after 5000 days (Figure 4: 35 vs. 41%), and a small decrease in the number of uninfected Tx cells. The overall number of Tx cells remained relatively constant in number. As shown in Figure 3, a reduction in shRNA efficacy not only increased the number of Tx cells infected with wt virus (0.1 vs. 6%), but also increased the number of cells containing resistant strains (m = 1; 0.0058 vs. 0.142%). The number of infected UNTx cells was unaffected by a decrease in shRNA efficacy and the resistance profile within this compartment remained constant. Overall, a reduction in shRNA efficacy increased the expansion of cells containing resistant virus by > 1 log, but only caused a small increase in the total number of infected cells after 5000 days (35 - 41%).

### Modeling changes in number of gene-containing cells

The transduction, reinfusion and engraftment of autologous HSC generate a population of CD4+ T cells in the periphery that contains the integrated gene therapeutic [16,17]. While current protocols can effectively transduce 20 - 50% HSC, the number of reconstituted circulating CD4+ T cells derived from transduced HSC (in the absence of initial marrow ablation) has been demonstrated to be no greater than 1% [21]. We therefore assessed the impact of a reduced number of gene-containing HSC from 20% to an apparently more biologically relevant 1%.

A reduction in the proportion of HSC containing 6 shRNAs from 20% (S1) to 1% (S5) increased the number of infected cells from 35 to 42% after 5000 days (Figure 3). However, for each of these scenarios, the total number of Tx cells, of which 99% were uninfected, was still greater than the total number of infected cells. Increased infection was caused by an increase in infected UNTx cells (Table 2: S1 & S5). This is in direct contrast to the increase in the number of infected cells as a result of a decrease in shRNA efficacy, which was due to the expansion of Tx cells containing resistant virus (Figure 4: S1). Reduced gene-containing HSC did not alter the resistant profile of virus in either UNTx or Tx compartments (Table 2). This is likely due to the survival advantage of cells that are adequately protected by 6 shRNAs. However, inadequate protection did alter the expansion of each cellular compartment and the resistance profile as a result of reduced marking. For example, cells with 2 shRNAs were more rapidly infected (Figure 3: S6 & S7, reaching 73.5 - 81.9%). This was due to a small decrease in the number of Tx cells (36 - 33%) including a decline in uninfected Tx cells (26 vs. 18%) and a simultaneous increase in the number of UNTx cells infected with wt virus (Table 2: S6 & S8).

### Modeling changes in viral fitness

Viral fitness refers to the overall capacity of the virus to replicate and is an important factor in explaining different resistant patterns to treatment [27,28]. We assessed the impact of decreased viral fitness for mutated viruses of 99% and 90%, as well as 50% for each mutation, regardless of its position. Where scenarios provided adequate protection (e.g. 4 or more shRNAs), a decrease in viral fitness did not have any major effect on overall infection or resistance profiles (Table 2: S1, S8 & S9). In all cases, uninfected Tx cells suppressed infection, as demonstrated by S1 (Figure 4). Infected cells accumulated when there was inadequate protection, e.g. in 2 shRNA (Figure 4: S3), and changes in viral fitness had no impact on this process (Table 2: S3, S10 & S6). However, a reduction in fitness did impact on the resistance profile in the UNTx and Tx compartments for combinations of 2 shRNAs (Figure 3).

### Treatment efficacy

The containment of resistance to the gene therapy is only one measure of total efficacy. Further measures of therapy effectiveness can be obtained by the extent of viral suppression as measured by the proportion of uninfected cells. With no gene therapy or when it only acts as a Class II agent, almost all cells quickly become infected (Figures 2A, 3F, Table 2 S11). With 4 and 6 shRNAs the proportion of infected cells was limited to approximately 40% over the entire 5,000 days. Even poorly suppressive therapies with only 2 shRNA resulted in significantly lower levels of infected cells for extended periods (Figure 2D).

### Gene therapy Class

The scenarios simulated thus far in this study have assumed that each shRNA exhibits both Class I and Class II levels of inhibition. We further used our model to assess the importance of inhibiting the incoming virus by removing the Class I component and found that cells became rapidly infected (Figure 4: S11). Almost all of the infected cells harboured wt virus (98%) and the majority of these cells were UNTx (90%). Removing the Class I component also increased the number of cells containing resistant virus (Figure 3: S11). The contribution of the Class I component of the shRNA produced an infected cell profile not significantly different to the scenario when no treatment was applied.

## Discussion

The model developed here is the first to simulate HIV infection within a 3-dimensional matrix, and study the efficacy of multiple shRNA gene therapies delivered by HSC. Recent evidence indicates that infection through direct cell contact, as occurs within lymphoid tissue, can occur via several mechanisms and may be a primary route of infection [29,30]. The model presented studies a mixed population of Tx and UNTx cells to mimic *in vivo *gene therapy conditions and mirrors the establishment of Tx CD4+ T cells in the periphery after engraftment of gene-containing HSC. Thus the proportion of Tx CD4+ T cells develops over time, rather than being at a fixed level from the start of therapy. This approach is similar to *in vitro *and *in vivo *studies that aim to mimic a mixed population of Tx and UNTx cells to the development of HIV resistance [31,32] and is in contrast to others which pre-select cells to ensure 100% of cells contain the gene therapeutic prior to infection [6,33]. Ideally, as done here, such studies should assess the development of resistance in a mixed population of cells in order to increase the biological relevance and better predict the dynamics of potential resistance in gene therapy.

Assuming that each shRNA was stably expressed in all Tx cells, the model shows that an increasing number of shRNAs provides greater efficacy and prevents the selection of escape mutants. Within the bounds of the assumptions contained in our model, this work predicts that a therapy comprised of 2 shRNAs results in a poor outcome with a high proportion of Tx cells infected and the emergence of mutated resistant virus. Increasing the number of shRNAs to 4 improved overall efficacy, which was increased even further with 6 shRNAs. This model does not account for the potential for virus to mutate non-protein target sites as a mechanism to compensate for antiviral activity as has been demonstrated by others [31]. Any model is dependent on the assumptions and while the number of shRNA needed cannot be exactly determined, there is strong support for the concept that sufficient shRNA (here represented by at least 4 shRNA) will provide efficacy without developing resistance in the same manner to HAART.

It is relevant that simultaneous expression of 4 shRNA has previously been shown to provide durable suppression of HIV in *in silico *models that do not consider Class I components [22] and *in vitro *models [34]. It is also relevant that in contrast to HAART, even when shRNA are insufficient to suppress viral replication (here represented by 2 shRNA), the failure to suppress replication will not drive the development of resistance. Importantly, the model shows that the inhibition of incoming virus is critical to effective therapy which has been indicated by others, albeit in deterministic models [19]. This model supports *in vitro *studies assessing primary HSC-derived macrophages by Anderson *et al*., which demonstrate importance of blocking incoming virus [35].

Although gene therapy is limited by the degree of expansion of transduced cells in this simplified model of lymphoid tissue, it can still provide a measure of effectiveness against viral replication and hence of CD4+ T cell depletion. Our simulations indicate that a 20% transduction of HSC can eventually translate into a much greater suppression of infection in the periphery. In our calculations 4 and 6 shRNA reduced infection levels by 60%. This added effect is due to the survival advantage of the transduced CD4+ T cells provided the gene therapy acts as a Class I agent. Even an inferior therapy containing 2 shRNA suppressed infection for extended periods of time (Figure 2D).

The population size (343,000 cells) was chosen to ensure i) that low frequency events could be meaningfully quantified, including the evolution of randomly mutated strains occurring in the absence of gene therapy and ii) consistency of results. As a validation of the model, it is relevant that variations in assumptions between scenarios produced quantitative results in the expected ordering of percentage resistant mutations and the variation over the 10 simulations for each scenario were small (Table 2). Nevertheless the complexity of the problem and the significantly smaller number of cells simulated *in silico *compared to the approximately 10^8 ^to 10^9 ^infected cells in an individual [36] suggest our results are indicative of the different situation for gene therapy compared to systemic antiretroviral therapy.

Not only is HIV established in short-lived activated CD4+ T cells, but it also infects resting CD4+ T cells, monocytes and macrophages and creates a latent pool. These other cell types can produce virus over lengthy life-spans and latently infected cells in particular exhibit the history of infection evolution within the individual. They are also strongly implicated in re-establishing high viral levels after the cessation of antiretroviral therapy. Hence a realistic model of HIV infection should duplicate i) infection not being in all target cells, ii) infection being maintained even at low levels, iii) and long-lived infected cells stopping eradication of virus even when infection is reduced to very low levels. Our model was designed to replicate these properties and the results presented show that it achieves these goals.

The inclusion of long-lived infected cells in the model was necessary in achieving these properties, as is expected in vivo as well. If the model only included short-lived infected cells then infection in the absence of the gene therapy either swamped the entire population or was completely extinguished. Moreover the addition of the gene therapeutic also either completely extinguished the infection, or established itself in all cells due to the high turnover with extensive infection. None of these situations duplicated what is expected to occur in practice and so models consisting solely of short-lived cells were discarded. It is interesting that the inclusion of a long-lived infected cell component allowed a better model of HIV infection both in the presence and absence of gene therapy. Although long-lived infected cells play an important role in vivo their half-lives have been estimated to be between 2 weeks and 44 months [36,37], and are expected to exhibit lower viral production. In that case the long-run level of infected cells will be expected to be less given that by the end of the simulations virtually all of the infected cells were long-lived. However, even with these limitations the model provided outcomes that are reasonable. With 2 shRNA, infection outgrows this poorly suppressive therapy and resides in both transduced and untransduced cells (Figure 2D). This inferior therapy also provides little pressure to develop resistance (Table 2). Simulation of more effective therapy with 4 shRNA constrains infection to a greater degree but is less effective than 6 shRNA (Figure 2B,C).

The model assumed that every infected cell that died was replaced by a new cell from the thymus or by one of its neighbors regardless of its phenotype, and if selective pressure is high, this results in Tx cells quickly becoming the dominant population. While this assumption and outcome are conservative, its consequence is that the selection pressure for resistant virus was even greater than would be expected. Further, it was assumed that each shRNA inhibited virus by 80% compared with wt, and that the effect of each additional shRNA was multiplicative irrespective of the presence of other shRNAs. For example, 6 shRNAs exhibited a 99.994% efficacy. However, multiple short interfering RNAs (siRNAs) and shRNAs may compete with each other and with host miRNAs for access to the RNAi machinery and therefore may not inhibit their targets as effectively as if they were expressed independently at maximal levels [2,38-42]. Future models may benefit from incorporating a diminishing return for each extra shRNA in order to model this scenario [22]. Conversely, competitive effects may be mitigated by using sub-saturating expression levels, as others have reported increased suppressive activity from multiple shRNAs [6,43,44].

Our own experience, as well as that of others, in gene therapy delivered to HSC and/or directly to CD4+ T cells, indicates that an anti-HIV gene therapy will not lead directly to the development of an entire CD4+ T cell population containing the therapeutic gene(s). It will likely be contained within a minority of these cells. Hence gene therapy provides a very different scenario to systemic antiretroviral therapy where every cell is bathed in some concentration of drugs. Our model was also designed to duplicate this situation where there should always be a sizeable proportion of target cells that do not contain the gene therapeutic. Given that this would present a situation very different to systemic therapy our model was designed specifically with this in mind, and it also achieves this goal.

The model was designed to follow cells, their survival and their replacement longitudinally through multiple rounds of possible infection. Unlike the model of Leonard [22], it allowed us to analyze the relative contribution of the gene therapy inhibiting incoming virus (Class I) and/or nascent viral transcripts (Class II). In all but one simulation, shRNAs were assumed to protect equally against Class I and Class II. While it is clear that RNAi can suppress HIV replication, there is conflicting opinion on whether it acts on the incoming genome, newly made viral transcripts, or both. Several groups have reported degradation of incoming RNA using siRNAs and shRNAs [25,26], whereas others have reported the opposite [45-47]. Westerhout *et al. *[48] studied this in detail and suggested that the incoming virion core is not completely disassembled and may be shielded from access by the RNA Induced Silencing Complex. This is an important point, since our modelling showed that targeting the incoming viral genome is critical for treatment success. If shRNAs are unable to target incoming RNA, then our model predicts that they must be combined with another technology that has Class I inhibition, such as peptide entry inhibitors (e.g. C46) [49-51].

## Conclusions

**Figure 5:**
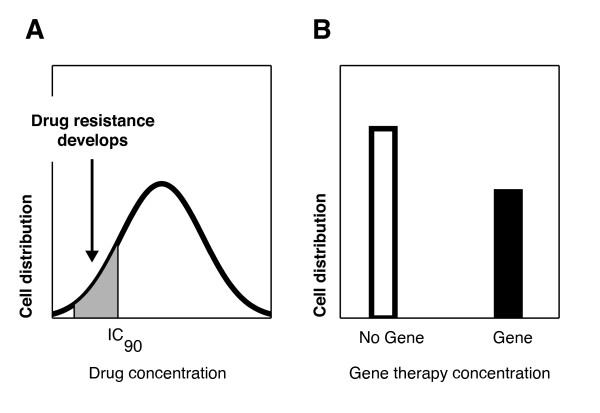
**Selection pressures driving the development of resistance**. The selection pressures driving resistance in (A) systemic antiretroviral therapy, compared to (B) the bipartite distribution of gene therapy.

In summary, resistance to gene therapy appears to differ from that for antiretroviral therapy. Although HSC gene therapy aims to establish a large protected population of target CD4+ T cells, monocytes and macrophages with the gene therapeutic, there will always be a proportion of UNTx target cells, particularly in the absence of complete bone marrow ablation. Thus there will be two distinct populations of target cells; UNTx cells which have no selective pressure driving the evolution of virus, and Tx cells which have the inhibitory pressure of gene therapy limiting viral replication. This differs from systemic antiretroviral therapy where cells contain a continuous distribution of the inhibitory effects of therapy and therefore provide a spectrum of selection from the outset (Figure 5). Thus, in the antiretroviral case, many cells will be only partly protected at suboptimal therapeutic concentrations, allowing viral replication and preferential development of drug-resistant strains.

Failure of gene therapy will occur if there is sufficient replication of virus within Tx cells and if the fitness of the resistant virus remains competitive with wild-type in UNTx cells. This situation is exemplified by cases where the gene therapeutic is suboptimal, allowing preferential expansion and eventual outgrowth of virus within Tx cells. This is shown by scenarios where the gene therapeutic contains 2 shRNA and generates outgrowth of virus regardless of the fitness of resistant virus. However, the modelling shows that viruses containing mutations that confer as little as a 5% and 1% loss in fitness are rapidly outcompeted by the wt in the UNTx cell compartment. This predicts that resistant viruses harbouring a reduction in fitness will not survive in the presence of an adequate gene therapy. The dynamics of virus outgrowth will be determined by i) efficacy of the gene therapeutic inhibiting wt replication in Tx cells, ii) fitness of resistant virus allowing it to replicate in UNTx cells in competition with wt; and iii) the relative sizes of the Tx and UNTx compartments. It will thus be important to develop a gene therapeutic to which resistant viruses have significantly reduced fitness, and that provides maximum inhibition by targeting incoming virus prior to integration. As it is presently unclear whether anti-HIV shRNAs alone can achieve this, multiple shRNA gene therapies may need to be combined with a Class I inhibitor to allow Tx cells to survive and mitigate the effects of HIV.

## Methods

This stochastic model of gene therapy for HIV incorporated the introduction of multiple anti-HIV shRNA into HSC cells which then differentiated into gene-containing progeny CD4+ T cells (referred to as Tx in the model). This model generated a longitudinal description of the expansion of both Tx cells, and infected cells, while monitoring the development of resistance. While the model incorporates some features similar to the model developed by Leonard *et al. *[22], it models transduction of HSC rather than mature lymphocytes and utilizes a large number of HIV target cells at all stages to incorporate the likelihood of resistant viral strains containing single and double mutations. Unlike Leonard, it does not assume 75-100% gene-marked T cells at baseline.

CD4+ T cells were assumed organised on the grid points of a 70 × 70 × 70 3-dimensional lattice in order to roughly duplicate the 3-dimensional layout of CD4+ T cells in a portion of lymphoid tissue. Initially all cells were uninfected and untransduced. The time step in these calculations was taken to be 12 hours.

Infection was established on this lattice by randomly choosing cells with a probability of 0.05 to become infected with wild-type virus. This initial infection pre-transduction was run for 200 time steps to generate viral diversity. Within each time step of this initial phase as well as the later post-transduction phase, cells died, were replaced by new cells from the HSC or through cellular division of a neighbouring cell, or were infected by neighbour cells. Each of these components is described in detail below.

Transduction with the gene therapy was assumed to establish a proportion of HSC that produced this same proportion of new CD4+ T cells. However, initially no cells on the lattice were transduced. Starting with the distribution of infection established in the initial phase, the model was run over 10,000 time steps, resulting in simulations over 5,000 days.

At the final time point, the proportion of cells on the lattice that were infected and/or transduced was calculated. Furthermore we determined the proportion of cells that were totally resistant to 1, 2, 3, ... of the shRNA components. Means and standard deviations over 10 simulations for each scenario are reported.

### Transduction

HSC were assumed transduced with a set number of shRNA. Simulations were performed where was either 2, 4 or 6 in separate experiments. Each shRNA target site was assumed to be 19 nucleotides (nt) long. Model parameters and their values are summarised in Table 3.

**Table 3:** Parameter descriptions

Parameters	Definition	Values
N	Number of shRNA transduced into HSC	2, 4, or 6
P	Proportion of transduced HSC	1%, 20%
Time - step	Time-step of each iteration	12 hours
eff	Efficacy of each shRNA inhibiting viral production and/or infection, dependent on resistance mutations. Inhibition per shRNA was (1-eff).	0.8, 0.5, 0
fitness	Fitness of viral strain relative to wild type	0.5, 0.9, 0.99
lifespan	Lifespan of newly infected cell. Determined from Poisson distribution. Cells with a lifespan of 0 are considered long-lived.	Mean value of 4 time-steps.
P.neigh	Probability a dead cell is replaced by an uninfected neighbour, provided one exists. Alternatively it is replaced by a new cell generated from HSC.	0.99
P.mut	Probability of mutation per nt per reverse transcription	3.4 × 10^-5^
P.inf	Probability of infection relative to total neighbouring viral production and cell transduction	0.0667

### Calculations during each time-step

#### Infected cells

Upon infection a cell was assigned a lifespan from a Poisson distribution with a mean of 4 time-steps (2 days [52]). Those cells assigned a 0 time-step lifespan (probability 0.0183) were considered long-lived infected cells, and remained alive and infected for the duration of the simulation. At each time-step infected cells were aged by one time-step and those that had reached their lifespan were removed. Uninfected cells were not aged. For this and later processes a neighbour of a cell is defined to be one of the 6 cells directly connected to it on the lattice, that is cells North or South, East or West, front or back. The model considered the 2 main processes by which Tx HSC cells give rise to Tx CD4+ T cells: direct export from the thymus and peripheral expansion of previously exported Tx CD4+ T cells (Figure 1A). This maintained a constant number of cells during each simulation. A dead infected cell was replaced by either a new cell exiting from the thymus and originating from HSC (1 - P.neigh), or was replaced through cell division of an uninfected neighbouring cell (P.neigh). CD4+ T cell division was given a much greater probability (than new cells exiting from the thymus) since naive CD4+ T cells are believed to be predominantly generated through peripheral expansion due to considerable involution of the thymus in adults [53,54]. If the dead cell was replaced by a new cell from the thymus then the probability this was transduced was given by the level of transduction in the HSC. Through this replacement process transduced CD4+ T cells could be seeded in the lattice, and could expand peripherally.

#### Viral sequence

Each viral genome was described in terms of the presence or absence (1 or 0) of mutations in each of the 19 nucleotide positions for each of the shRNA. A mutation in the 7 central nucleotides resulted in complete resistance to that particular shRNA ***(eff = 0)***. A mutation in the remaining regions provided partial resistance ***(eff = 0.5)***. Any 2 mutations among the 19 nt rendered the shRNA completely ineffective.

### Viral production by an infected cell

Each infected cell produced virions at a rate dependent on the sequence of its integrated viral genome and whether it was transduced or not. An untransduced infected cell produced virions at a standardised rate of 1. For an infected transduced cell containing ***N ***shRNA with no resistance mutations, viral production was scaled down by a factor of ***(1-eff)^N^***. If the infected cell contained a partial or fully resistant mutation to a single shRNA, then the efficacy of the shRNA to which the virus sequence contained the mutation was reduced from 80% to 50% or 0% respectively. Hence a cell infected with a viral strain totally resistant to all shRNA would have the same viral productivity, modulo fitness, as an untransduced cell. Viral production was multiplied by a fitness factor if the viral genome was not wild-type.

It was assumed that all shRNAs inhibited viral production (Class II) but depending on the scenario simulated could also inhibit incoming viral transcripts (Class I) at the same rate [55]. This inhibition was assumed to be multiplicative so that if single shRNAs were 80% efficacious (i.e. inhibited replication to 20% of that seen with UNTx cells) then 2 shRNAs inhibited replication to 4% (0.2^2^), and so forth.

### Infection of a cell

If an uninfected cell possessed infected neighbours then the chance it became infected per time step was determined by the viral production of these cells, the transduction status of the uninfected cell, the viral sequence of the infecting virions, and chance. Viral production from neighbouring infected cells was calculated as described above.

1. If the uninfected cell was untransduced then viral production over all neighbouring cells was summed, and multiplied by a scaling factor ***P. inf***. The resulting value was taken to be the mean of a Poisson distribution. The random number generated from this distribution and for the uninfected cell determined the number of virions that could infect the cell. Multiple infecting virions allowed the possibility of recombination and further viral diversity. The infecting virions were then randomly chosen over all neighbours relative to their viral productivity. The sequences of each of these infecting virions were used as the basis for mutation and recombination as described below.

2. If the uninfected cell was transduced, and if the shRNA were allowed to inhibit infection and act as a Class 1 gene therapy, then the viral productivity of each infected neighbour was modified by the inhibition by the shRNA in the uninfected cell relative to the particular viral sequence. The efficacy of inhibition was assumed equivalent to the efficacy of viral production as described above. This calculation took into account the viral sequence of each of the infected neighbours. The calculations then proceeded as above. The choice of the infecting virions was also based on this modified viral production for each cell.

### Mutation and recombination

Due to the lack of a proof-reading mechanism, HIV replication (like all retroviruses) is error prone and characterized by a high mutation rate. HIV sequence variation was randomly incorporated at a rate of 3.4 × 10^-5 ^mutations/nt/reverse transcription (P.mut) in accord with the estimated error rate for the HIV-1 reverse transcriptase (RT) [23]. If a target cell was likely to be exposed to more than 1 virion, then the infecting virions were randomly assigned between 0 and 3 recombination events (over a full 6 × 19 viral sequence relevant to the shRNA) in accord with the recombination rates predicted for HIV [56], further increasing viral sequence diversity. The nt positions where the RT enzyme jumps to the other infecting virion were chosen from a discrete uniform distribution. The viral genome sequence for the infected cell was the resulting mutated and recombined sequence. The scenarios modelled are shown in Table 1.

## Competing interests

The authors declare that they have no competing interests.

## Authors' contributions

TLA, GLM, JMM and DJB conceived and designed the model. JMM wrote and developed the mathematical model. All authors participated in data analysis and interpretation. TLA, GJM and JMM prepared the manuscript.
